# Redox modifications of cysteine residues regulate the cytokine activity of HMGB1

**DOI:** 10.1186/s10020-021-00307-1

**Published:** 2021-06-07

**Authors:** Huan Yang, Peter Lundbäck, Lars Ottosson, Helena Erlandsson-Harris, Emilie Venereau, Marco E. Bianchi, Yousef Al-Abed, Ulf Andersson, Kevin J. Tracey

**Affiliations:** 1 Laboratory of Biomedical Science, The Feinstein Institute for Medical Research, Manhasset, NY USA; 2 Department of Medicine, Center for Molecular Medicine, Karolinska Institutet, Karolinska University Hospital, Stockholm, Sweden; 3 Department of Women’s and Children’s Health, Karolinska Institutet, Stockholm, Sweden; 4 Vita-Salute San Raffaele University, Milan, Italy; 5 Division of Genetics and Cell Biology, Chromatin Dynamics Unit, IRCCS San Raffaele Scientific Institute, Milan, Italy; 6 Center for Molecular Innovation, Feinstein Institutes for Medical Research, Northwell Health, Manhasset, NY USA

**Keywords:** HMGB1, Isoforms, TLR4, Redox, Cytokine, Receptor, Inflammation

## Abstract

**Background:**

High mobility group box 1 (HMGB1) is a nuclear protein with extracellular inflammatory cytokine activity. It is passively released during cell death and secreted by activated cells of many lineages. HMGB1 contains three conserved redox-sensitive cysteine residues: cysteines in position 23 and 45 (C23 and C45) can form an intramolecular disulfide bond, whereas C106 is unpaired and is essential for the interaction with Toll-Like Receptor (TLR) 4. However, a comprehensive characterization of the dynamic redox states of each cysteine residue and of their impacts on innate immune responses is lacking.

**Methods:**

Primary human macrophages or murine macrophage-like RAW 264.7 cells were activated in cell cultures by redox-modified or point-mutated (C45A) recombinant HMGB1 preparations or by lipopolysaccharide (*E. coli*.0111: B4). Cellular phosphorylated NF-κB p65 subunit and subsequent TNF-α release were quantified by commercial enzyme-linked immunosorbent assays.

**Results:**

Cell cultures with primary human macrophages and RAW 264.7 cells demonstrated that fully reduced HMGB1 with all three cysteines expressing thiol side chains failed to generate phosphorylated NF-КB p65 subunit or TNF-α. Mild oxidation forming a C23-C45 disulfide bond, while leaving C106 with a thiol group, was required for HMGB1 to induce phosphorylated NF-КB p65 subunit and TNF-α production. The importance of a C23–C45 disulfide bond was confirmed by mutation of C45 to C45A HMGB1, which abolished the ability for cytokine induction. Further oxidation of the disulfide isoform also inactivated HMGB1.

**Conclusions:**

These results reveal critical post-translational redox mechanisms that control the proinflammatory activity of HMGB1 and its inactivation during inflammation.

## Introduction

This report replaces a study (Yang et al. 2012) that was retracted (Yang et al. 2020) because mass-spectrometry data provided by one of the authors had been fabricated. The present report, where the fabricated data were removed, reaffirms the functional information reported at the time, and can be correctly cited as the first evidence of redox-sensitive properties of HMGB1 regarding the ability of the molecule to promote inflammation.

High mobility group box-1 protein (HMGB1), a 25 kDa nuclear protein with 99 % identity among mammals (Sessa and Bianchi 2007), has been implicated as a mediator of tissue damage and inflammation during sterile injury and infection (Andersson and Rauvala 2011). It is released passively during cell injury and is secreted actively by macrophages and other cell types activated by exposure to products of infection or injury. Extracellular HMGB1 signals via TLR4 to activate macrophages to release TNF-α, IL-1, IL-6 and other proinflammatory molecules (Yang et al. 2010). Neutralizing HMGB1 antibodies reverse inflammation and attenuate disease severity in animal models (Andersson and Tracey 2011; Schierbeck et al. 2011; Wang et al. 1999).

HMGB1 has three major protein domains consisting of two tandem DNA-binding regions (box A and box B), and an acidic carboxyl terminus comprised of a string of aspartate and glutamate residues (Bianchi et al. 1989). Initial studies of the structural basis for the extracellular proinflammatory cytokine activity of HMGB1 revealed a critical role for residues in the B box domain (Li et al. 2003). The first 20 amino acid residues (position 89–108) of the B box domain represent the minimal peptide sequence that retains cytokine-inducing activity by macrophages (Li et al. 2003). The activity of HMGB1 to stimulate macrophage cytokine production requires HMGB1 binding to TLR4, and both binding and signaling require the cysteine in position 106 (C106) within the B box domain (Yang et al. 2010).

The induction of immune responses is critically intertwined with a dynamic redox environment, and posttranslational redox modifications represent key mechanisms to regulate protein functions. Understanding the functional relationship between redox modifications of key inflammatory extracellular signaling proteins, such as HMGB1, is thus of great importance. As a redox-sensitive protein, HMGB1 contains three cysteine residues: C23, C45, and C106. Recently, it was suggested that oxidation of C106 modulates the activity of HMGB1 and prevents its capacity to activate dendritic cells (Kazama et al. 2008). While the focus to date on redox remodeling has been on C106, there are two additional conserved cysteine residues present within HMGB1 (C23 and C45). The structure:function relationship between modifications on these residues to the inflammatory activity of HMGB1 is unknown. The formation of an intramolecular disulfide bond between C23 and C45 stabilizes the folded state of the full-length protein and generates a conformation change (Sahu et al. 2008) that might impact upon the signaling function of HMGB1 as an inflammatory mediator (Sahu et al. 2008; Stott et al. 2010). The present study was thus performed to study posttranslational redox mechanisms controlling the cytokine-inducing activity of HMGB1. The results indicate that the inflammatory activities of HMGB1 require a fully reduced C106 and a formation of a disulfide bond between C23 and C45.

## Materials and methods

### Materials

Lipopolysaccharide (LPS, *E. coli*.0111: B4), triton X-114 and human macrophage-colony stimulating factor (M-CSF) were purchased from Sigma (St. Louis, MO, USA). Isopropyl-D-thiogalactopyranoside (IPTG) was from Pierce (Rockford, IL, USA). DNase I and 2xYT medium were obtained from Life Technologies (Grand Island, NY, USA). Hydrogen peroxide (3 %) was from Fisher Scientific (Waltham, MA, USA). Dithiothreitol (DTT, 100 mmol/L) was obtained from Invitrogen (Carlsbad, CA, USA). HMGB1 enzyme-linked immunosorbent assay (ELISA) kit was purchased from Shino-Test Corporation (Tokyo, Japan).

### Generation of recombinant and mutant HMGB1 proteins

Wild-type rat *HMGB1* was cloned, expressed and purified as described previously. To generate C45A mutant HMGB1, the cysteine at position 45 in the wild-type HMGB1 clone was substituted with alanine using the QuikChange, Site-Directed Mutagenesis kit according to the manufacturer’s instructions (Stratagene, Skarholmen, Sweden). The primers used were, forward: 5′-GAG TTC TCC AAG AAG GCC TCA GAG AGG TGG AAG ACC-3′ and reverse: 5′-GGT CTT CCA CCT CTC TGA GGC CTT CTT GGA GAA CTC-3′. The PCR product was subcloned into the pCAL-n vector, 3′ to the T7 promotor and CBP tag, expressed and purified as described previously (Yang et al. 2010). DNase I was added at 100 U/mL to the beads to remove any contaminating DNA. Degradation of DNA was verified by ethidium bromide staining of agarose gel containing HMGB1 proteins before and after DNase I treatment. The purity and integrity of purified HMGB1 proteins was verified by Coomassie Blue staining after SDS-PAGE, with purity predominantly above 90 %. Contaminating LPS from protein preparations was removed by Triton X-114 extraction as described previously (Li et al. 2004). LPS content in the protein preparations was typically less than 0.1 EU/µg protein by the LAL method.

### Preparation of redox-modified HMGB1 or mercury-exposed HMGB1

HMGB1 was exposed to either hydrogen peroxide (H_2_O_2_; 0–50 µmol/L) or dithiothreitol (DTT) (0–5 mmol/L) for up to 2 h prior to the addition of the protein to cell culture or other analysis. For rHMGB1 that had been produced in the presence of DTT, an exposure of 50 µmol/L H_2_O_2_ for 2 h was utilized. In order to expose HMGB1 to mercury, the HMGB1 solution was treated with 1:1 or 1:10 molar equivalents of 4-(hydroxyl-mercury) benzoic sodium salt. The reaction was stirred at room temperature for 2 h and excess mercury was subsequently removed by dialysis against PBS at 4 °C overnight.

### Cell cultures

Peripheral blood mononuclear cells were isolated from blood of healthy volunteers (Long Island Blood Services, Melville, NY, USA) and differentiated into macrophages using M-CSF. Murine macrophage-like RAW 264.7 cells were obtained from American Type Culture Collection (Rockville, MD, USA). Cells were cultured in 96 or 6 well plates as described previously (2).

### Cytokine measurements

TNF-α released in the supernatants of macrophage cultures were measured by commercially available enzyme-linked immunosorbent assay (ELISA) kits according to instructions of the manufacturer (R&D Systems Inc., Minneapolis, MN, USA).

### Measurements of cellular phosphorylated NF-κB p65 subunit expression

Phosphorylated NF-κB p65 measurement in primary human macrophages was performed using RayBio NF-kB p65 ELISA kit (Cat #PEL-NFKBP65-S536-T) according to the instructions (RayBiotech, Norcross, GA). Total cell lysate was prepared and used in the assay as per manufacturer’s instructions.

### Statistical analysis

Data are presented as mean ± SEM. Differences between treatment groups were determined by Student *t* test; one-way ANOVA followed by Tukey’s multiple comparison tests or Two-way ANOVA followed by Sidak’s multiple comparisons test between groups. *P* values less than 0.05 were considered statistically significant by using the Graphpad Prism software.

## Results

### HMGB1-induced TNF-α production requires a disulfide bond between C23 and C45 and C106 expressing a thiol side chain

The three cysteines present in HMGB1 are strictly conserved in all mammals (Sessa and Bianchi 2007). Previous studies established that C106 is required for HMGB1-induced cytokine release, since a point mutation of C106 prevented the binding to TLR4/MD2 and subsequent cytokine release from activated macrophages/monocytes (Yang et al. 2010). Other studies indicated that an intramolecular disulfide bond between C23 and C45 generates a conformation change in the full-length protein (Sahu et al. 2008) and that HMGB1 oxidation may impact its inflammatory properties (Kazama et al. 2008). However, the structure-function relationship of the three cysteine residues and the cytokine-inducing activity of HMGB1 remains to be clarified.

**Fig. 1:**
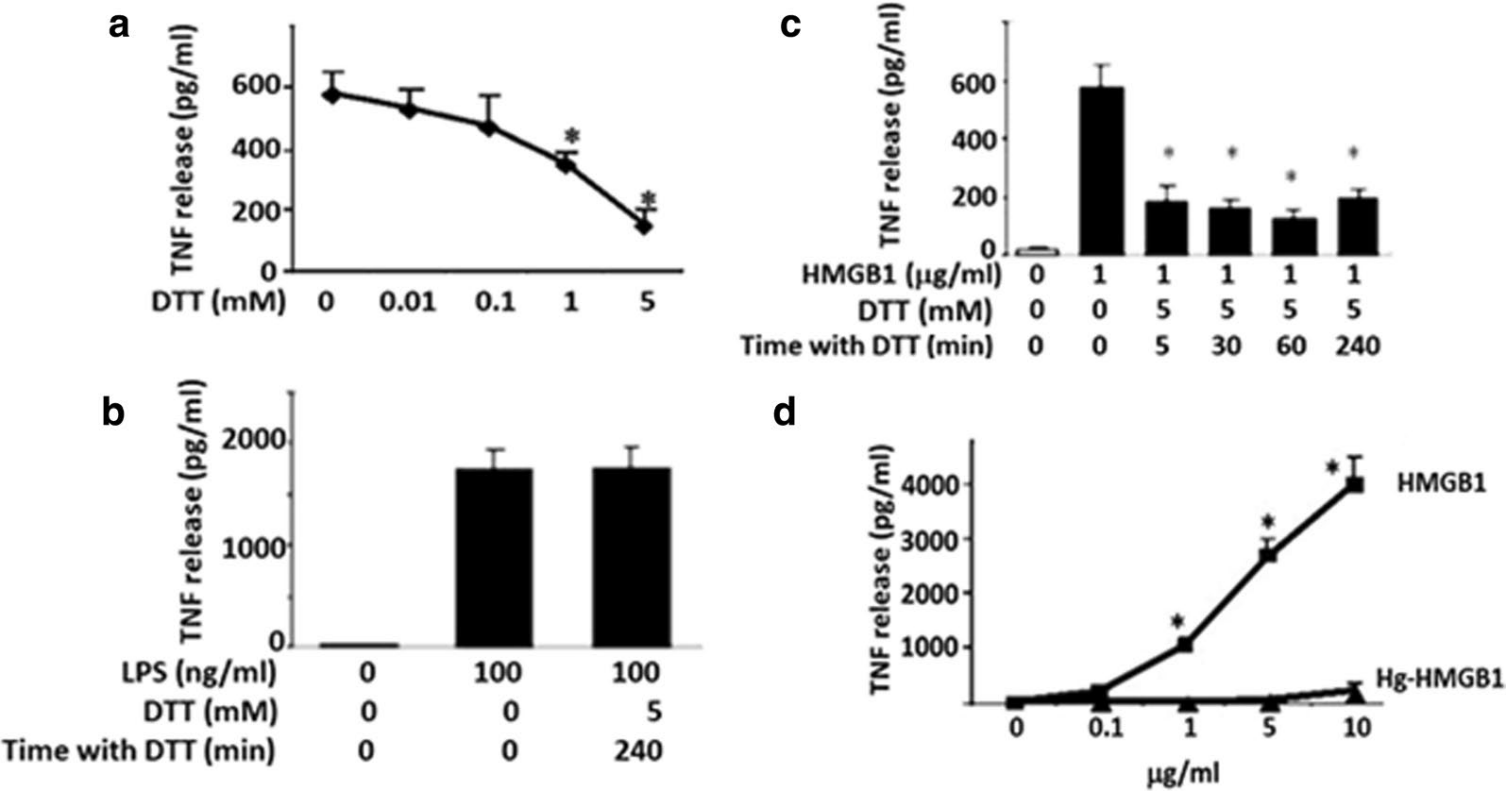
The cytokine-stimulating activity of HMGB1 requires the presence of a disulfide bond between C23-C45 and the C106 residue in its fully reduced form. **a** Dose-dependent effect of DTT on the TNF-α-stimulating activity of HMGB1 (1 µg/ml, 16 h incubation), indicating that DTT prevented the formation of a disulfide bridge between C23 and C45. *P < 0.05 versus HMGB1 alone without DTT. N = 3. One-way ANOVA followed by Tukey’s multiple comparisons tests. **b** Lack of DTT-mediated effect on LPS regarding its TNF-α-stimulating activity. **c** Time-dependent effect of DTT on the TNF-α-stimulating activity of HMGB1. One-way ANOVA followed by Tukey’s multiple comparisons test between groups. *p < 0.05 versus HMGB1 without DTT. **d** The effect of mercury (Hg) on TNF-α release induced by recombinant HMGB1 prepared in the absence of DTT. Mercury selectively binds to thiol side chains. The results implicate that out of the 3 cysteines expressed in HMGB1 it must be C106 carrying a thiol side chain in TNF-α-inducing HMGB1, since C23 and C45 are engaged in a disulfide bond. n = 3. Two-way ANOVA followed by Sidak’s multiple comparisons test between groups. *p < 0.05 versus Hg-HMGB1

We started the investigation of cysteine redox requirements by studying the influence of reducing conditions on the cytokine-inducing performance of HMGB1. The reducing agent dithiothreitol (DTT) prevents and reverses intra- and intermolecular disulfide bond formation between cysteine residues. RAW 264.7 cells were co-cultured with DTT-exposed or non-exposed HMGB1 and the subsequent TNF-α release was dose-dependently reduced by DTT (Fig. 1a). Addition of DTT did not significantly alter LPS-induced TNF-α release in the cultures supporting that the effects by DTT on HMGB1 were specific (Fig. 1b). Time course studies revealed that incubation of HMGB1 with DTT for only 5 min suppressed HMGB1 activity as a TNF-α stimulator by up to 70 % (Fig. 1c). We thus concluded that DTT prevented the formation of a disulfide bridge between C23 and C45 that in turn inhibited TNF-α release.

We further investigated the redox state of the cytokine-inducing HMGB1 by exposing highly purified recombinant HMGB1 to mercury (Hg) to elucidate whether C106 expressed a thiol side chain or not when HMGB1 activated RAW 264.7 cells to release TNF-α. Mercury selectively reacts with the thiol side chain of a cysteine, but not with other cysteine redox forms. Mercury exposure significantly reduced TNF-α release in Hg-HMGB1 stimulated cultures as compared to non-Hg exposed cultures (Fig. 1d). These results thus demonstrated that the critical C106 residue must be in its fully reduced form expressing a thiol side chain to activate TNF-α release. Taken together these findings indicated that reduced C106 alone was insufficient for HMGB1-mediated TNF-α induction and demonstrated that the redox states of all three cysteines contributed to the TNF-α-stimulating activity of HMGB1. The results implied that the cytokine-inducing redox form of HMGB1 has a need of a disulfide bond between C23 and C45 alongside with C106 expressing a thiol side chain (C23-S-S-C45, C106-SH) (Fig. 1d).

### Effects of cysteine oxidation on the cytokine-stimulating ability of HMGB1

**Fig. 2:**
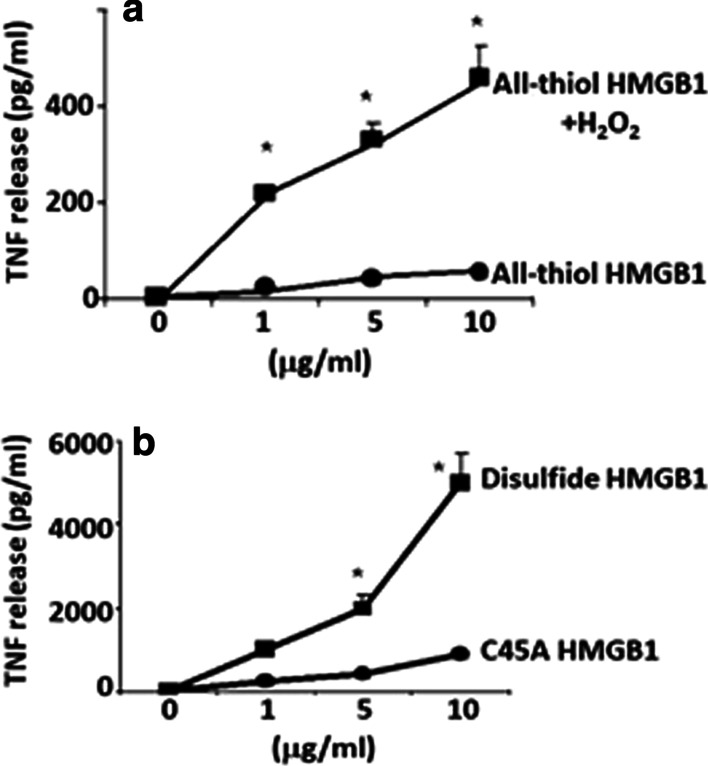
Effect of oxidation on HMGB1-induced TNF-α release in RAW 264.7 cells. **a** Effects of mild H_2_O_2_ exposure (50 µmol/L, 120 min) on the TNF-α stimulating activity of HMGB1 prepared with DTT (all-thiol HMGB1). **b** Effects of an A45 mutation of HMGB1 on TNF-α release compared with HMGB1 prepared in the absence of DTT (disulfide HMGB1). RAW 264.7 cells were incubated with various HMGB1 proteins over a range of concentrations (0 to 10 µg/mL) for 16 h. TNF-α released into the cell culture supernatant was measured by ELISA. N = 3. Two-way ANOVA followed by Sidak’s multiple comparisons test between groups. *p < 0.05 versus all-thiol or C45A-HMGB1

To further examine the redox state of the cytokine-inducing isoform of HMGB1 we exposed the molecule to various intensities of oxidation. As expected, no TNF-α release occurred in RAW 264.7 cell cultures stimulated by fully reduced HMGB1 (C23-SH, C45-SH, C106-SH) prepared in the presence of DTT (Fig. 2a). However, a strong TNF-α release was observed in cultures stimulated by the DTT-prepared HMGB1 that had been exposed to mild oxidation caused by hydrogen peroxide (H_2_O_2_ 50 µM, 120 min) (Fig. 2a). A mild oxidation should support a formation of the C23-C45 disulfide bond. The restored ability of the DTT-produced HMGB1 to activate cytokine production after mild oxidation is in line with the notion that the cytokine-inducing redox isoform of HMGB1 expresses a disulfide bridge between C23 and C45 with C106 in the thiol form.

The importance of a C23–C45 disulfide bond for HMGB1-mediated cytokine production was confirmed by mutation of C45 to an alanine in the full length HMGB1 protein. This point mutation prevents the formation of a disulfide bond between C23 and C45. We observed that the C45A HMGB1 mutant had significantly reduced TNF-α-stimulating activity in RAW 264.7 cells compared to the C45-expressing HMGB1 protein (Fig. 2B).

**Fig. 3:**
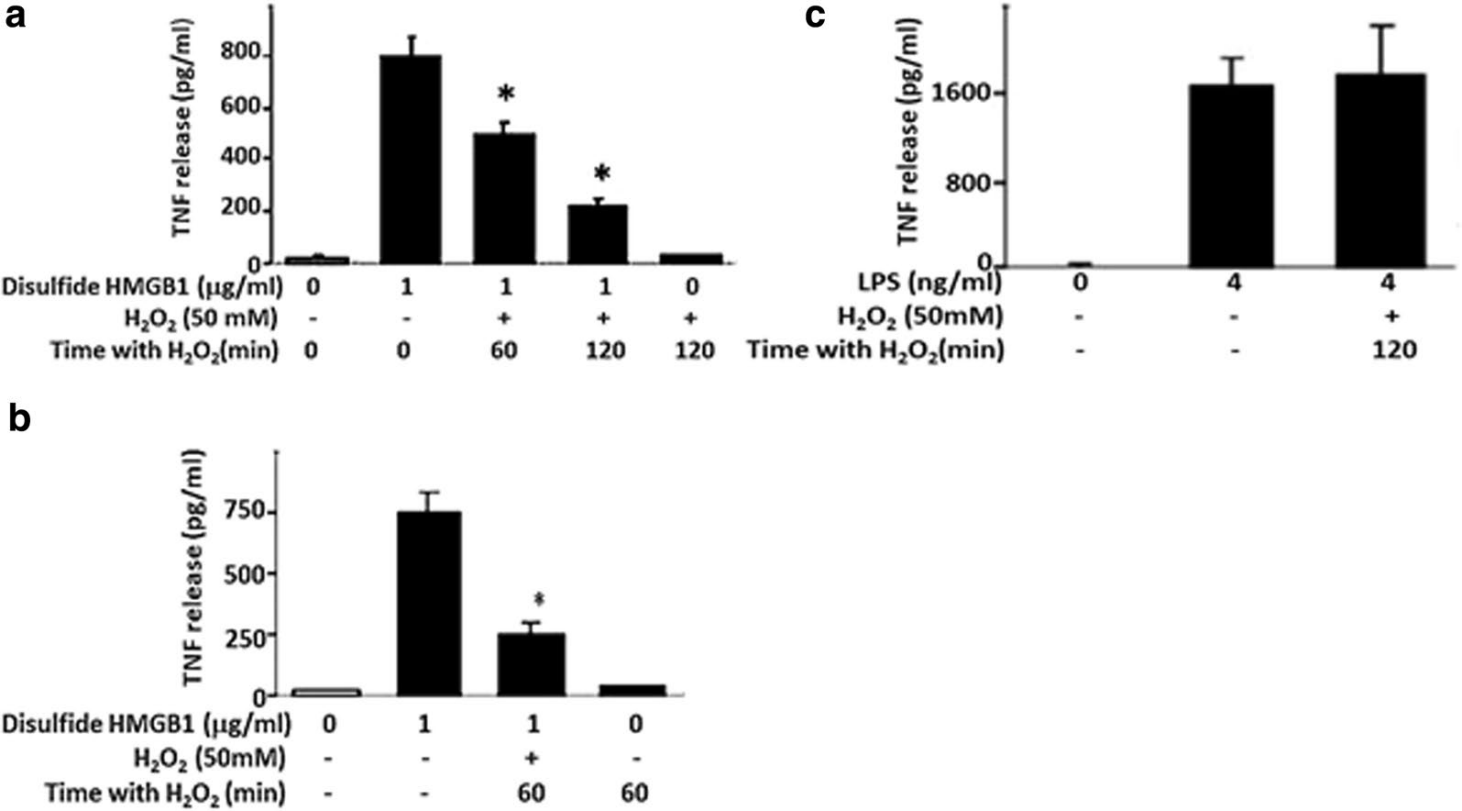
The time-dependent inhibitory effect of H_2_O_2_ exposure on the TNF-α-stimulating activity of HMGB1 in **a** RAW 267.7 cells and **b** primary human macrophages. **c** The effect of H_2_O_2_ exposure on the TNF-α-stimulating activity of LPS in RAW 264.7 cells. Cells were cultured in 96-well plates and stimulated with HMGB1 or LPS with or without exposure to 50 mM H_2_O_2_ for 0–120 min. Number of experiments in each panel: n = 3. One-way ANOVA followed by Tukey’s multiple comparisons test between groups. *p < 0.05 versus HMGB1

We then exposed cytokine-inducing HMGB1 to robust oxidation by hydrogen peroxide (50mM). The strongly oxidized HMGB1 lost its capacity to induce TNF-α in cell cultures with RAW 264.7 cells (Fig. 3a) as well as with human primary macrophages (Fig. 3b). Hydrogen peroxide-exposure of LPS failed to inhibit TNF-α-inducing activity, indicating that the effect of hydrogen peroxide was HMGB1 specific (Fig. 3c). In summary, these results reinforce the identification of disulfide HMGB1 as the cytokine-inducing isoform.

### Disulfide HMGB1 induces NF-kB p65 subunit formation

**Fig. 4:**
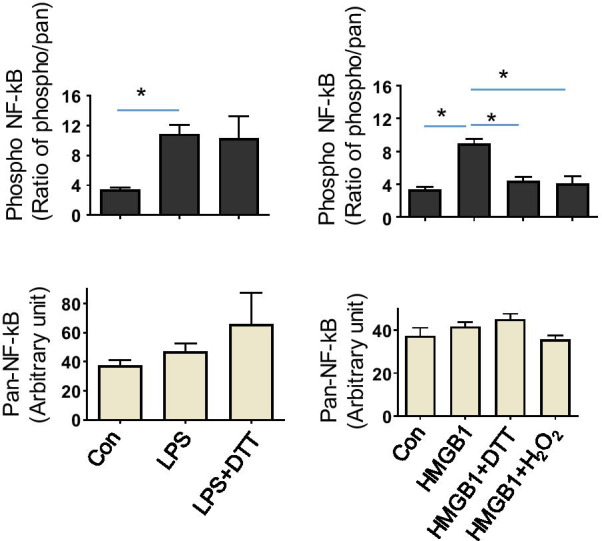
Redox-dependent effect on HMGB1-induced NF-κB activation in primary human macrophages. Analysis of phosphorylated NF-κB p65 subunit following the stimulation of cultured macrophages in 6-well plate for 1 h with HMGB1 (5 µg/mL) or LPS (4 ng/mL). HMGB1 was exposed to either H_2_O_2_ (50 mM, 120 min) or DTT (5 mM, 120 min) prior to the assay. LPS was exposed to DTT (5 mM, 120 min). Expression of phosphorylated NF-κB p65 and total NF-κB p65 (loading control) was measured by quantitative ELISA. Data are presented as ratio of phospho-NF-κB vs. total NF-κB or means ± SEM. n = 3. *p < 0.05. One-way ANOVA followed by Tukey’s multiple comparisons test between groups

We further studied redox-dependent effects on HMGB1-induced NF-kB p65 subunit activation in primary human macrophages, a process required to initiate the production of many proinflammatory cytokines. The cytokine-inducing disulfide HMGB1 isoform promoted a phosphorylation of NF-kB p65, while DTT-exposed HMGB1 and strongly oxidized HMGB1 did not (Fig. 4). These results were thus in line with the observed requirements for HMGB1-induced TNF-α release being dependent on disulfide HMGB1.

## Discussion

Oxidative modification represents a key post-translational alteration to regulate the functional capacity of signaling proteins. Much is known about the functional modulation of intracellular proteins such as enzymes (phosphatases and kinases) and transcriptional regulators through the chemo-selective oxidation of cysteine residues (Spickett and Pitt 2012). Cysteine redox perturbation is also thought to alter the extracellular functionality of intercellular signaling proteins (Rubartelli and Lotze 2007). During tissue inflammation and repair, the changing redox environment following cell death can have important consequences on the bioactivity of key inflammatory signaling proteins (Vezzoli et al. 2010).

The aim of the current investigation was to provide a chemical definition of the pattern of cysteine redox modifications regulating the cytokine-inducing capacity of HMGB1. We conclude that to induce cytokine release, HMGB1 must simultaneously have C106 in its reduced thiol form and C23 and C45 engaged in a disulfide bridge. Robust oxidation or full reduction to thiol groups each inactivates HMGB1. The modifications induced by robust oxidation have not been studied by us and remain an important issue to elucidate in future studies.

The most common covalent linkages between protein side chains are disulfide bonds, and disulfide-thiol interconversions alter protein properties through changes in chemical reactivity and conformation. These redox-dependent changes will thereby regulate many critical functions of signaling proteins including HMGB1 (Hogg 2003; Park and Lippard 2011). C23 and C45 readily form a disulfide bond (Hoppe et al. 2006) which increases the stability of the folded full length HMGB1 molecule; NMR analysis confirms that this linkage alters the conformation of the A domain within the HMGB1 protein (Park and Lippard 2011; Sahu et al. 2008). C23 and C45 mutations prevent HMGB1 from binding to the intracellular target protein Beclin 1, inhibiting HMGB1 from initiating cellular autophagy processes (Tang et al. 2010). Mild oxidation of completely reduced HMGB1, which is unable to induce cytokine formation, partially restored the C23–C45 disulfide bond and the cytokine-inducing capacity. These findings provide a proof of concept for the critical role of the C23-S-S-C45 linkage. Surface plasmon resonance-based studies, reported several years after our observations outlined here by us, confirm that the box A and box B domains of HMGB1 both are both needed for proper interaction with the TLR4/MD-2 receptor, although with different binding and kinetic parameters. The box A domain binds to TLR 4 with high affinity and slow off-rate, while once in close proximity the box B domain binds to MD-2 with low affinity but extremely slow off-rate (He et al. 2018). Furthermore, recent studies in tissue samples obtained from pathological and physiological conditions underline a close association of disulfide HMGB1 expression with an inflammatory state characterized by the presence of immune cells and identifies leukocytes as reservoirs of disulfide HMGB1 (Ferrara et al. 2020; Kwak et al. 2020).

The unpaired cysteine C106 may theoretically form a disulfide bridge with another C106 residue on a second HMGB1 molecule which might have functional consequences. Dimeric HMGB1 is an alternative chemical entity which has not been investigated by us and remains a subject for future studies.

Acetylation of key lysine residues within the nuclear localization sequence of HMGB1 is thought to act as a key regulatory mechanism to promote a nuclear export enabling further extracellular release of HMGB1 from macrophages and monocytes, and extracellular acetylated HMGB1 has been confirmed *in vivo* (Bonaldi et al. 2003). Acetylation of nearby lysine residues might change the electrostatic potential and affect the pK_a_ of cysteine thiol groups; however, as previously noted (Sahu et al. 2008), no lysine amino group is present within 8 Å from the thiol groups in the three-dimensional structure of the HMGB1, and therefore an impact on the regulation of the proinflammatory function of HMGB1 is unlikely.

Since the original description of HMGB1 activity in stimulating macrophage cytokine release (Andersson et al. 2000), investigations have revealed that HMGB1 can act in synergy with other inflammatory mediators to promote cytokine stimulation (Hreggvidsdottir et al. 2009). Therefore, there has been an ongoing debate concerning whether HMGB1 *per se*, uncomplexed to partner molecules, is capable of inducing cytokine production to promote inflammation. The results of the current study help to resolve this major issue: HMGB1 with reduced C106 and a disulfide bond between C23 and C45 has cytokine-stimulating activity, whereas many commercially available rHMGB1 preparations, being supplemented with DTT and fully reduced, are unable to stimulate TNF-α production, but highly competent of promoting chemotactic activities (Schiraldi et al. 2012).

## Conclusions

In summary, the results provided here reveal a novel mechanism to regulate the activities of a key signaling protein, HMGB1, and suggest that HMGB1 might be a central target of redox modifications during inflammation and its resolution.

## Data Availability

All data supporting the findings of this study are available within the paper.
